# Syntaxin 11 Contributes to the Interferon-Inducible Restriction of Coxiella burnetii Intracellular Infection

**DOI:** 10.1128/mbio.03545-22

**Published:** 2023-02-02

**Authors:** Sandhya Ganesan, Natalie N. Alvarez, Samuel Steiner, Karen M. Fowler, Abigail K. Corona, Craig R. Roy

**Affiliations:** a Department of Microbial Pathogenesis, Yale University School of Medicine, New Haven, Connecticut, USA; b Department of Pediatrics, School of Medicine, Emory University, Atlanta, Georgia, USA; c Department of Immunobiology, Yale University School of Medicine, New Haven, Connecticut, USA; Ohio State University

**Keywords:** *Coxiella*, innate immunity, interferons, intracellular bacteria, phagosomes, secretion systems

## Abstract

There is a limited understanding of host defense mechanisms targeting intracellular pathogens that proliferate in a lysosome. Coxiella burnetii is a model bacterial pathogen capable of replicating in the hydrolytic and acidic environment of the lysosome. It has been shown that gamma interferon (IFNγ)-stimulated host cells restrict C. burnetii replication by a mechanism that involves host IDO1 depletion of tryptophan. Host cells deficient in IDO1 activity, however, retain the ability to restrict C. burnetii replication when stimulated with IFNγ, which suggests additional mechanisms of host defense. This study identified syntaxin 11 (STX11) as a host protein that contributes to IFNγ-mediated suppression of C. burnetii replication. STX11 is a SNARE protein; SNARE proteins are proteins that mediate fusion of host vesicles with specific subcellular organelles. Depletion of STX11 using either small interfering RNA (siRNA)- or CRISPR-based approaches enhanced C. burnetii replication intracellularly. Stable expression of STX11 reduced C. burnetii replication in epithelial cells and macrophages, which indicates that this STX11-dependent cell-autonomous response is operational in multiple cell types and can function independently of other IFNγ-induced factors. Fluorescently tagged STX11 localized to the *Coxiella*-containing vacuole (CCV), and STX11 restriction was found to involve an interaction with STX8. Thus, STX11 regulates a vesicle fusion pathway that limits replication of this intracellular pathogen in a lysosome-derived organelle.

## INTRODUCTION

Activation of mammalian cells with the cytokine gamma interferon (IFNγ) restricts the replication of most intracellular pathogens and promotes clearance of the pathogen from the host. This is achieved by production of IFNγ-regulated proteins that promote cell autonomous defense mechanisms that ultimately lead to the destruction of the pathogen in a lysosome (1). Little is known about how IFNγ activation promotes host defense against specialized pathogens that have evolved to survive and replicate inside lysosome-derived organelles. To address this question, we are investigating the mechanisms of IFNγ-mediated restriction of Coxiella burnetii intracellular replication.

Coxiella burnetii is an obligate intracellular bacterial pathogen that replicates in an acidified lysosome-derived vacuole referred to as the *Coxiella*-containing vacuole (CCV). The C. burnetii *dot* and *icm* genes encode a type IVB secretion system (T4SS) that is essential for biogenesis of a CCV that supports intracellular replication (2, 3). C. burnetii encodes over 100 different proteins called effectors that are translocated into the host cytosol by the Dot/Icm system, and these effector proteins mediate the subversion of multiple host cell pathways (4–13). Effector proteins have been shown to promote the remodeling and expansion of the CCV by controlling fusion with autophagic, secretory, and clathrin-coated vesicles (8, 14–20).

Host Ras-associated binding (RAB) GTPases promote endocytic maturation and progressive acidification of the nascent CCV, which delivers C. burnetii to an acidified lysosomal organelle (21). SNARE proteins (Soluble N-ethylmaleimide-sensitive factor Attachment protein REceptor) associated with the CCV membrane promote fusion of the CCV with host vesicles and organelles through cognate SNARE-SNARE interaction on the opposing membranes. Mammals encode roughly 36 different SNARE proteins, and the pairing of cognate SNARE proteins regulates organelle-specific delivery of cargo in membrane-bound vesicles (22–25). To generate the autolysosomal organelle in which C. burnetii replicates, the SNARE protein VAMP7 has been shown to promote fusion of the CCV with lysosomes (26), and the endoplasmic reticulum (ER)-localized SNARE protein syntaxin 17 (STX17) mediates fusion of the CCV with autophagosomes (8, 27–29).

The cytokine IFNγ is critical for host protection against pathogens that have evolved the ability to replicate in acidic and hydrolytic lysosomal compartments (30–35). Many hundreds of different genes are upregulated when cells are activated by IFNγ; however, there remains much to learn about how these genes contribute to host defense. In human nonprofessional phagocytic cells, IFNγ-mediated restriction of C. burnetii replication is mediated primarily by upregulation of the host protein Indoleamine 2,3-dioxygenase 1 (IDO1), which is an enzyme that depletes tryptophan in the host cell to deprive C. burnetii of this essential nutrient (34). Silencing of *IDO1* partially rescues intracellular replication of C. burnetii in IFNγ-activated host cells, whereas inactivation of the IFNγ signaling pathway mediated by silencing of STAT1 completely restores C. burnetii replication (34). This indicates that other host factors contribute to IFNγ-mediated restriction of C. burnetii replication. The goal of this study was to identify additional IFNγ-regulated proteins that function in host restriction of C. burnetii replication.

## RESULTS

### STX11 contributes to IFNγ-mediated restriction of C. burnetii.

IDO1 is a dominant IFNγ-induced host factor that interferes with C. burnetii intracellular replication by depleting tryptophan (34). We hypothesized that IDO1 function may precede or mask the importance of other IFNγ-induced mechanisms that restrict C. burnetii replication. Thus, a small interfering RNA (siRNA) screen was conducted to identify host factors that contribute to IFNγ-mediated restriction of C. burnetii replication under conditions where the host cells were supplemented with additional tryptophan to override the effect of IDO1. Luminescence was measured to assess intracellular replication of a C. burnetii strain producing luciferase (as described in reference 34). Peak luminescence was used to compare the effect of gene silencing on C. burnetii replication in HeLa cells over 6 days of infection (see Fig. S1 in the supplemental material). Silencing of the gene encoding the SNARE protein Syntaxin 11 (STX11) resulted in a significant increase in C. burnetii luminescence in cells that were treated with IFNγ and supplemented with tryptophan (Fig. S1). These data suggested that STX11 may contribute to IFNγ-mediated restriction of C. burnetii intracellular replication (Fig. S1).

10.1128/mbio.03545-22.1 FIG S1 Syntaxin 11 (STX11) contributes to IFNγ-mediated restriction of C. burnetii. HeLa 229 cells were transfected with the indicated siRNA pools, infected with C. burnetii *lux*, and left untreated or treated with IFNγ in the presence of additional tryptophan (Trp). (A) *Coxiella* luminescence was measured at 4 dpi, and values of mock- or siRNA-transfected, IFNγ-treated cells (red bars) were normalized to that of mock-transfected cells that were not treated with IFNγ (blue bars). Values corresponding to that of mock-transfected, IFNγ-treated cells (second bar in each set) was set as the baseline for calculating statistical significance for the siRNA pools that exceeded the baseline. The screen was performed twice, and the average values are shown. Statistical significance was calculated by one-way ANOVA (A) with Bonferroni’s multiple-comparison test (*, *P* < 0.05; ***, *P* < 0.001; ****, *P* < 0.0001). Download FIG S1, TIF file, 1.7 MB. Copyright © 2023 Ganesan et al. 2023 Ganesan et al. https://creativecommons.org/licenses/by/4.0/ This content is distributed under the terms of the Creative Commons Attribution 4.0 International license.

**FIG 1:**
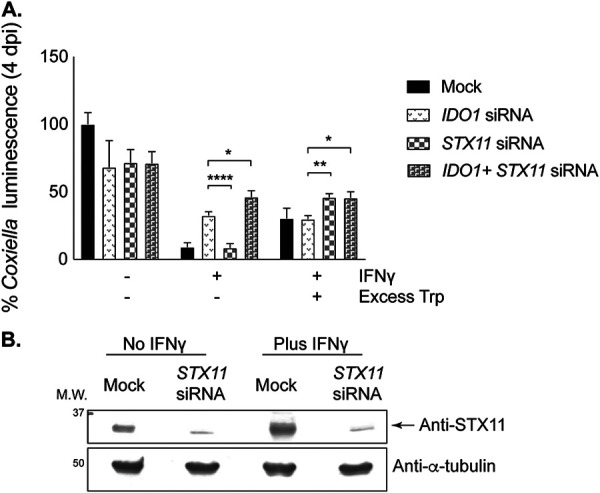
(A) Syntaxin 11 (STX11) contributes to IFNγ-mediated restriction of C. burnetii. HeLa 229 cells were transfected with the indicated siRNA pools, infected with C. burnetii *lux*, and left untreated or treated with IFNγ in the presence or absence of additional tryptophan (Trp). *Coxiella* luminescence data were normalized to those of mock-transfected, untreated cells (first black bar). For each treatment group, statistical significance was calculated by comparing the luminescence values of *IDO1* siRNA with those of *STX11* siRNA alone or *IDO1*+*STX11* siRNA. The experiment was conducted at least twice, and the average values are shown. (B) STX11 protein levels in mock- or *STX11* siRNA-treated cells left untreated or treated with IFNγ were examined by western blotting. Statistical significance was calculated by two-way ANOVA with Bonferroni’s multiple-comparison test (*, *P* < 0.05; **, *P* < 0.01; ****, *P* < 0.0001).

To validate the requirement of STX11 in IFNγ-mediated C. burnetii restriction, HeLa cells were treated with siRNA pools targeting either *IDO1* or *STX11* independently or pools that would silence both host factors (Fig. 1A). Consistent with the results in Fig. S1, silencing *STX11* in cells where *IDO1* was silenced (*IDO1*+*STX11* siRNA) and silencing *STX11* in Trp-supplemented cells significantly augmented C. burnetii luminescence in IFNγ-treated cells (Fig. 1A). Immunoblot analysis demonstrated production of the STX11 protein in HeLa cells, induction by IFNγ stimulation, and knockdown of *STX11* by siRNA pools in untreated and IFNγ-treated cells (Fig. 1B). Thus, neutralizing the effects of IDO1 was necessary to uncover a role for STX11 in IFNγ-mediated restriction of C. burnetii replication, which indicates a dominant phenotype for IDO1 in this defense pathway (Fig. 1).

### Cells deficient in STX11 expression are more permissive for C. burnetii intracellular replication.

**FIG 2:**
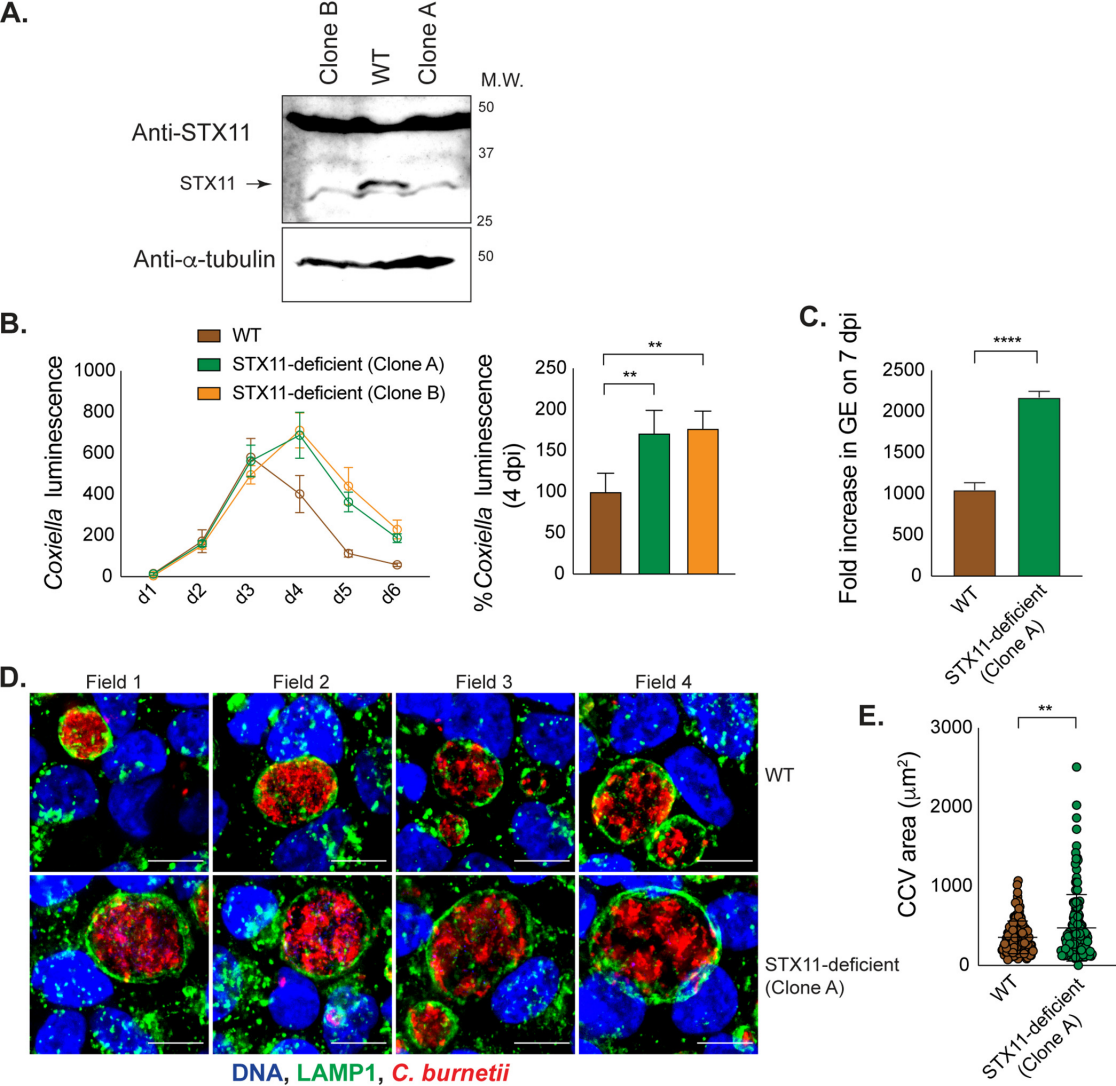
Cells deficient in STX11 expression are more permissive for intracellular *Coxiella* replication. (A) STX11 expression in clonal isolates of STX11-deficient HeLa CCL2 cells, as detected by western blotting. (B) STX11-deficient cells (clones A and B) and the corresponding wild-type control (WT) were infected with C. burnetii *lux*, and luminescence was measured on the indicated days postinfection. In the graph on the right, values measured on 4 dpi were normalized to that of the WT. (C to E) WT and STX11-deficient cells were infected with wt C. burnetii. (C) Fold increase in C. burnetii genome equivalents at 7 dpi compared to that at 1 dpi was measured by qPCR. (D) Infected HeLa cells stained for DNA, LAMP1, and C. burnetii were examined by indirect immunofluorescence on 5 dpi. Bars, 10 μm. (E) CCV area was measured for at least 100 vacuoles per cell type. Data in panels B, D, and E are representative of at least 3 experiments, and those in panel C are representative of at least 2 independent experiments. Statistical significance was calculated by one-way ANOVA (B) and unpaired *t* test (C and E) with Bonferroni’s multiple-comparison test where applicable (**, *P* < 0.01; ****, *P* < 0.0001), based on the biological replicates included in one representative experiment.

To better characterize STX11 function during C. burnetii infection, STX11-deficient HeLa cells were generated using CRISPR-based gene editing. HeLa cells constitutively expressing either *STX11* guide RNA (gRNA) or the corresponding empty vector were clonally isolated and designated STX11-deficient and WT* cells (WT HeLa that expresses plentiGuide-Puro empty vector), respectively (Fig. S2A). No STX11 protein was detected in the STX11-deficient HeLa cells. Replication of C. burnetii in the STX11-deficient cells was compared to replication in WT* cells. These data showed enhanced replication of C. burnetii in the STX11-deficient cells (Fig. S2B). Consistent with replication data based on luminescence, C. burnetii replication assessed by measuring genome equivalents (GE) revealed enhanced replication of C. burnetii in STX11-deficient cells compared to WT* cells (Fig. S2C). To validate the role of STX11 in restriction of C. burnetii replication and reduce off-target effects that could potentially arise due to constitutive expression of Cas9 and gRNA, STX11-deficient clones were isolated after transient expression of Cas9 and gRNA in HeLa CCL2 cells (Fig. 2A). Replication of C. burnetii was similarly enhanced in two independent clones of STX11-deficient HeLa CCL2 cells (Fig. 2B and C). CCVs in WT and STX11-deficient HeLa cells were immunostained for C. burnetii and a lysosomal membrane protein (LAMP1) and analyzed by fluorescence microscopy. CCVs stained positive for LAMP1 in the presence as well as the absence of STX11, indicating that STX11 is not required for maturation of the CCV to a lysosome-derived organelle. In the absence of IFNγ signaling, the average size of these LAMP1^+^ CCVs was increased in cells deficient in STX11 (Fig. 2D and E and Fig. S2D and E), consistent with data indicating enhanced bacterial replication in STX11-deficient cells (Fig. 2B and C and Fig. S2B and C). However, the small CCVs observed in IFNγ-treated cells displayed no significant difference in size (Fig. S2E), which is consistent with IDO1 having a dominant role in restricting C. burnetii replication. Altogether, these data suggest STX11 mediates a host defense pathway that restricts C. burnetii replication.

10.1128/mbio.03545-22.2 FIG S2 STX11 deficiency supports higher C. burnetii replication and CCV expansion. (A) HeLa 229 cells constitutively expressing Cas9 were transduced with empty vector or an *STX11* gRNA-expressing plasmid. Transduced cells were puromycin selected and clonally isolated for WT* and STX11-deficient cells, respectively. Expression of STX11 was detected in parental, Cas9-expressing, and empty vector-expressing but not *STX11* gRNA-expressing HeLa cells. STX11 was upregulated by IFNγ in all the STX11-expressing cell lines. (B) WT* and STX11-deficient cells were infected with C. burnetii *lux* and left untreated or treated with IFNγ 6 h postinfection. Bacterial luminescence was measured at 4 dpi and values normalized to that of untreated WT* cells. (C) WT* and STX11-deficient cells were infected with wt C. burnetii. Fold increase in C. burnetii genome equivalents at 7 dpi compared to that at 1 dpi was measured by qPCR. (D) WT* and STX11-deficient cells infected with wt C. burnetii were fixed and stained for DNA, LAMP1, and C. burnetii as indicated. CCVs were examined by fluorescence microscopy, and representative images are shown. (E) Average CCV area in WT* and STX11-deficient cells was measured under untreated and IFNγ-treated conditions. (F) GFP alone or GFP-STX11 was lentivirally transduced in parental wild-type HeLa 229 cells, and antibiotic selection was used to enrich for transduced cells. Cells were infected with C. burnetii *lux*, and bacterial luminescence at 3 dpi was normalized to that of GFP alone. Data in panels B to E are representative of two or more experiments. Statistical significance in panel B was determined by two-way ANOVA with Bonferroni’s multiple-comparison test, and that in panels C, E, and F was calculated by an unpaired *t* test (*, *P* < 0.05; **, *P* < 0.01; ****, *P* < 0.0001). Download FIG S2, TIF file, 5.7 MB. Copyright © 2023 Ganesan et al. 2023 Ganesan et al. https://creativecommons.org/licenses/by/4.0/ This content is distributed under the terms of the Creative Commons Attribution 4.0 International license.

### STX11 overexpression interferes with C. burnetii intracellular replication.

**FIG 3:**
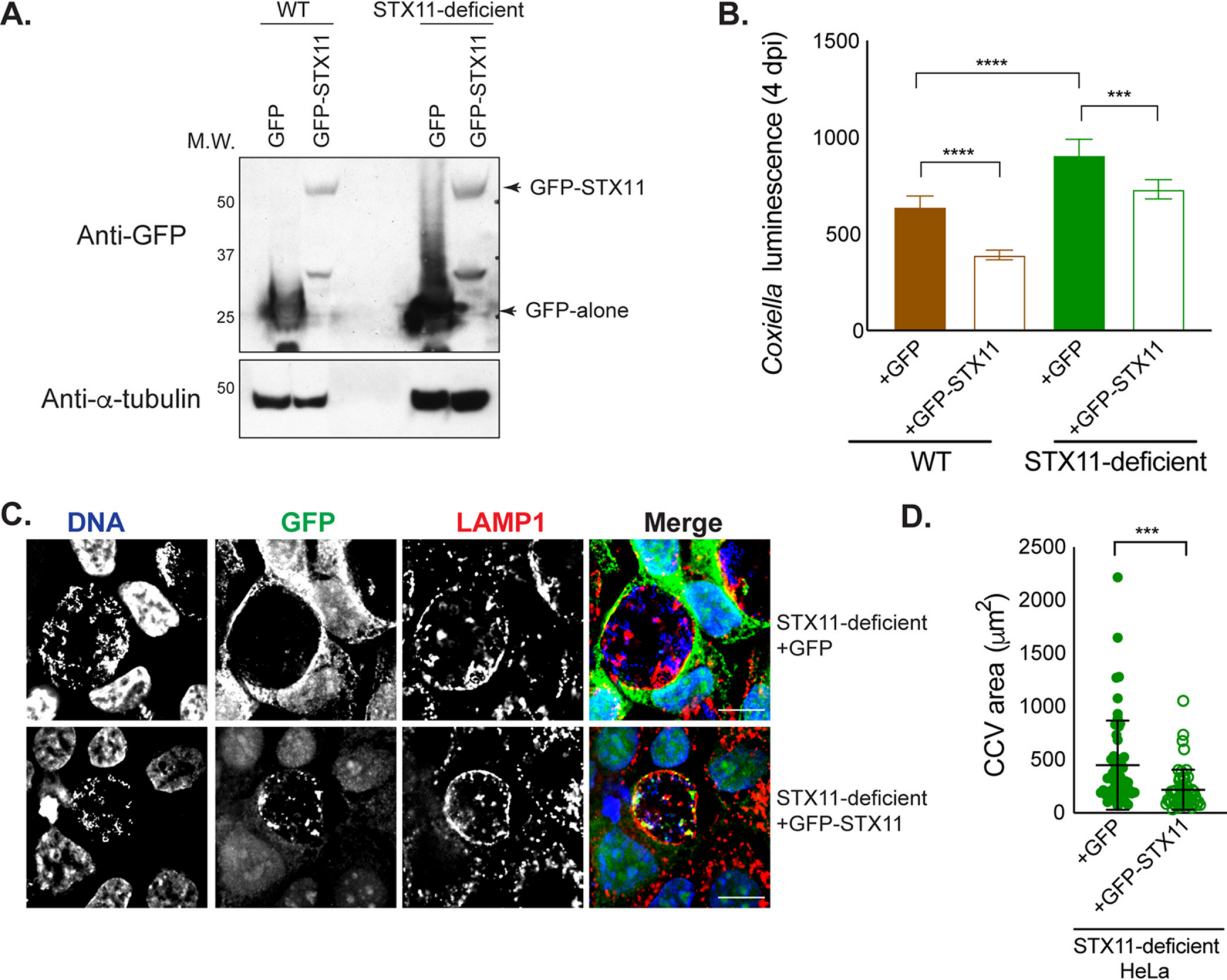
STX11 expression restricts C. burnetii. WT and STX11-deficient HeLa CCL2 cells were reconstituted with GFP-tagged STX11 or GFP alone. (A) Stable expression of GFP-STX11 and GFP was detected by Western blotting. (B) Transduced cells were infected with C. burnetii *lux*, and luminescence was measured at 4 dpi. Statistical significance was measured by one-way ANOVA with Bonferroni’s multiple-comparison test (***, *P* < 0.001; ****, *P* < 0.0001). (C) STX11-deficient cells reconstituted with GFP-STX11 or GFP alone were infected with wt C. burnetii. HeLa cells and CCVs stained for DNA, GFP, and LAMP1 were examined by indirect immunofluorescence at 4 dpi. One representative field containing CCV is shown. Bars, 10 μm. (D) CCV area was measured for at least 50 vacuoles per cell type. Data in panels B and D are representative of at least 3 or 2 independent experiments, respectively. Statistical analysis was calculated based on data obtained from one representative experiment, from the biological replicate wells (B) or vacuolar sizes from several fields (D) included in that experiment. Statistical significance was measured by Student’s *t* test (***, *P* < 0.001).

Green fluorescent protein (GFP)-tagged STX11 was produced ectopically in HeLa cells to determine if upregulation of STX11 would be sufficient to augment a host restriction pathway that limits C. burnetii intracellular replication. Immunoblot analysis confirmed the production of GFP-STX11 or GFP alone in the resulting stable cell lines (Fig. 3A). Production of GFP-STX11 decreased C. burnetii replication in both WT and STX11-deficient cell lines (Fig. 3B and Fig. S2F). This decrease in C. burnetii replication in cells producing GFP-STX11 correlated with a decrease in the average size of the CCV in these cells (Fig. 3C and D). These data indicate that increasing STX11 production augments restriction of C. burnetii intracellular replication, suggesting that STX11 protein levels may be a limiting factor regulating this cell autonomous defense pathway.

### STX11 associates with the mature CCV.

**FIG 4:**
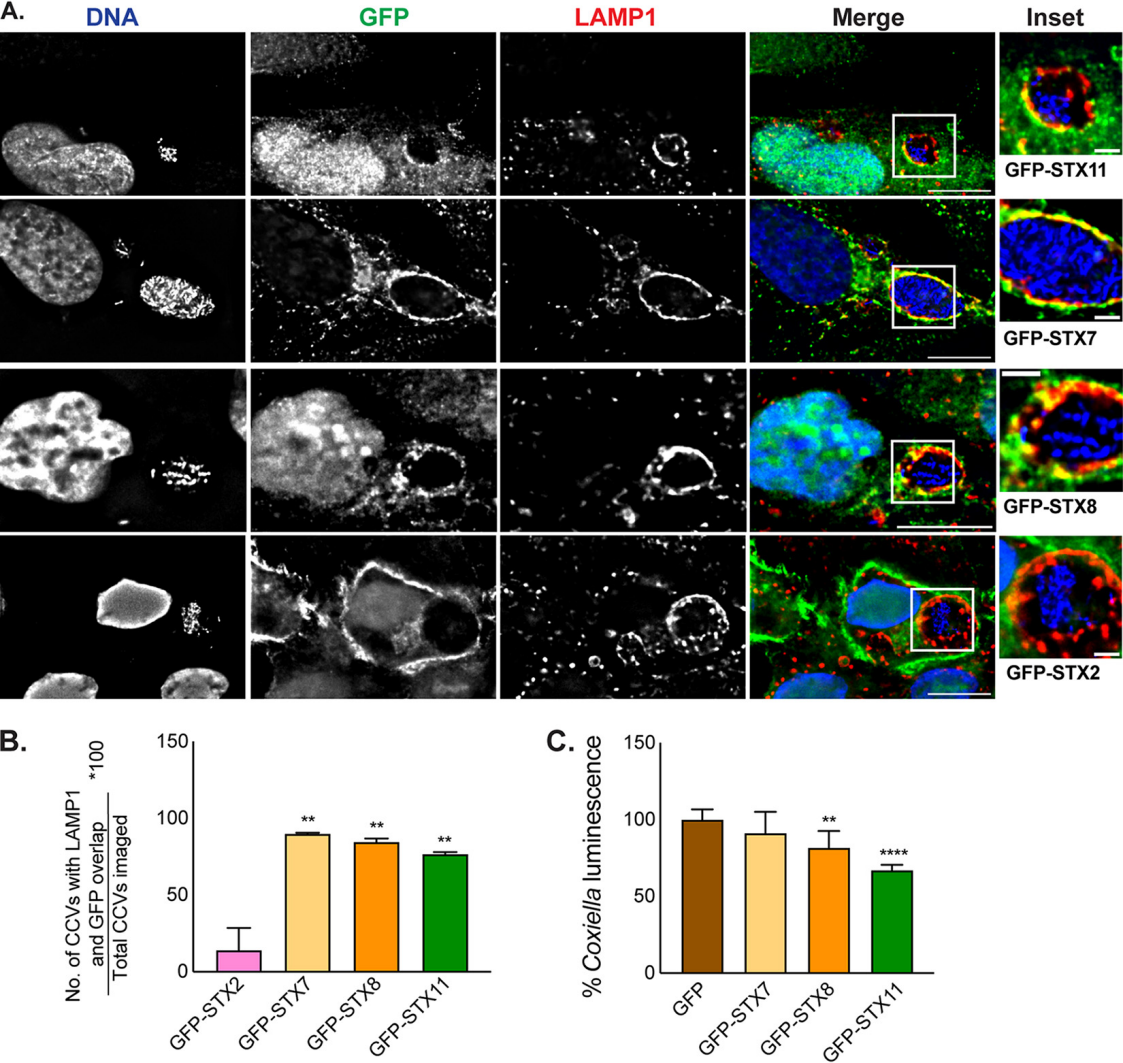
STX11 localizes to the CCV, and its expression specifically suppresses C. burnetii replication. HeLa cells expressing GFP-STX (STX11, -7, -8, and -2), as indicated, were infected with wt C. burnetii at MOI of 25 at 4 dpi, and CCVs were examined by indirect immunofluorescence for the markers shown (A). CCVs were examined for colocalization of GFP-STX with the CCV membrane using LAMP1 staining as the reference (B). Panel A depicts Representative examples of STX scored as positive (GFP-STX7, -8, and -11) and as negative (GFP-STX2) for the data in panel B. Number of CCVs that showed partial or complete overlap in LAMP1 and GFP staining on the boundary of the CCV, compared to the total number of CCVs imaged is shown in panel B. HeLa cells stably expressing GFP-alone or GFP-tagged STX7, STX8, or STX11 were infected with C. burnetii *lux*, and bacterial luminescence was measured at 4 dpi (C). Data in panels B and C are averages from two independent experiments. Statistical significance was determined using one-way ANOVA using Bonferroni analysis (**, *P* < 0.01; ****, *P* < 0.0001). Bars, 10 μm (A) and 2 μm (insets).

The localization of GFP-STX11 was examined in C. burnetii-infected cells to address whether STX11 could mediate the fusion of host vesicles with the CCV. Fluorescence microscopy was used to localize GFP-tagged proteins and vacuoles containing C. burnetii (Fig. 3C and Fig. 4A). Bacteria inside the CCV were identified by DNA staining, and the limiting membrane of the CCV was identified by staining for the host protein LAMP1. These micrographs demonstrate that GFP-STX11 localized to the limiting membrane of the CCV (Fig. 3C and Fig. 4A). Colocalization of GFP-STX11 with LAMP1 was evident on the vacuoles containing C. burnetii (Fig. 4A).

The localization of other SNARE proteins was examined to determine the specificity of GFP-STX11 localization to the CCV. STX7, STX8, and Vti1B are endosome-associated t-SNAREs (target membrane SNARE) that typically pair with the v-SNARE (vesicle membrane SNARE) VAMP8 or VAMP7 to drive fusion events with late endosomes or lysosomes, respectively (36, 37). Because these proteins are all involved in maturation of endocytic organelles, they should all have a role in CCV biogenesis. GFP-STX7 and GFP-STX8 were both found to be robustly associated with the CCV and colocalized with LAMP1 (Fig. 4A). STX2 is a plasma membrane-associated SNARE that may be transiently associated with an early CCV but is not predicted to play a direct role in CCV biogenesis. Consistent with this hypothesis, the mature CCV did not have STX2 associated with the limiting membrane, which demonstrates the selective association of host SNAREs with the CCV (Fig. 4A). Individual CCVs were analyzed and quantified for the association of these different SNARE proteins, which confirmed the association of STX7, STX8, and STX11 on the CCV and the absence of STX2 (Fig. 4B).

Because SNARE-SNARE interactions drive membrane fusion reactions, proteins in a complex with GFP-STX11 were identified by mass spectrometry. The GFP-STX11 protein was immunoprecipitated from uninfected and C. burnetii-infected cells. Consistent with previous studies identifying STX11 interacting proteins (38–41), the proteins SNAP23 and STXBP2 were identified in the GFP-STX11 complex (Fig. S3A). The STX8 protein was also identified in the GFP-STX11 immunoprecipitate, suggesting that STX8 could be a component of a complex containing STX11 (Fig. S3A). Immunoprecipitation of GFP-STX11 followed by immunoblotting for endogenous SNARE proteins demonstrated the association of STX8, VAMP8, and Vti1B in a complex with GFP-STX11 (Fig. S3B). Endogenous STX8 was detected on the CCV by fluorescence microscopy (Fig. S3C), consistent with previous observations (42, 43). HeLa cells expressed low levels of endogenous STX11, consistent with previous studies showing low expression of STX11 in nonlymphoid cells (38, 44, 45), which prevented specific detection of endogenous STX11 on the CCV in these cells. Overproduction of GFP-STX11 and GFP-STX8 after transient transfection of an expression plasmid enhanced localization of these GFP-tagged SNAREs to the CCV, providing further evidence that these proteins traffic to the CCV (Fig. S4).

10.1128/mbio.03545-22.3 FIG S3 STX8 interacts with STX11 and localizes to the CCV. Lysates from HeLa CCL2 cells stably expressing GFP alone or GFP-STX11 and uninfected or infected with C. burnetii NMII for 3 days were immunoprecipitated using anti-GFP antibody, on-bead digested using trypsin, and subjected to mass spectrometry (A). SNARE and syntaxin-binding proteins identified by spectral counts exclusive to these proteins in different samples are presented (A). Lysates and immunoprecipitates, as described for panel A, were immunoblotted for GFP, endogenous STX8, VAMP8, and Vti1B (B). Localization of endogenous STX8 in C. burnetii-infected cells at 3 dpi shown by indirect immunofluorescence (C). Long and short bars in the merged and inset images denote 10 μm and 2 μm, respectively (C). Download FIG S3, TIF file, 4.3 MB. Copyright © 2023 Ganesan et al. 2023 Ganesan et al. https://creativecommons.org/licenses/by/4.0/ This content is distributed under the terms of the Creative Commons Attribution 4.0 International license.

10.1128/mbio.03545-22.4 FIG S4 Transient transfection of GFP-STX11 or GFP-STX8 leads to enhanced localization to the CCV. HeLa CCL2 cells were transiently transfected with GFP alone (A) or GFP-tagged SNAREs, STX7 (B), STX11 (C), STX8 (D) and VAMP8 (E). Transfected cells were left uninfected or infected with mCherry-expressing C. burnetii and imaged live 3 dpi. Data are representative of at least 3 experiments. Bars, 10 μm (individual and merged channels) and 2 μm (insets). Download FIG S4, TIF file, 5.5 MB. Copyright © 2023 Ganesan et al. 2023 Ganesan et al. https://creativecommons.org/licenses/by/4.0/ This content is distributed under the terms of the Creative Commons Attribution 4.0 International license.

The interaction between STX11 and STX8 was unexpected, and suggested that a complex containing these two proteins may be involved in restriction of C. burnetii intracellular replication. Consistent with this hypothesis, ectopic production of GFP-STX8 interfered with C. burnetii replication similar to ectopic production of GFP-STX11 (Fig. 4C). In contrast, ectopic production of GFP-STX7, which also localized to the CCV (Fig. 4A), did not inhibit C. burnetii intracellular replication (Fig. 4C). These data suggest that STX8 and STX11 have a functional relationship in a pathway that restricts C. burnetii intracellular replication.

### STX11 expression interferes with C. burnetii replication in human macrophages.

**FIG 5:**
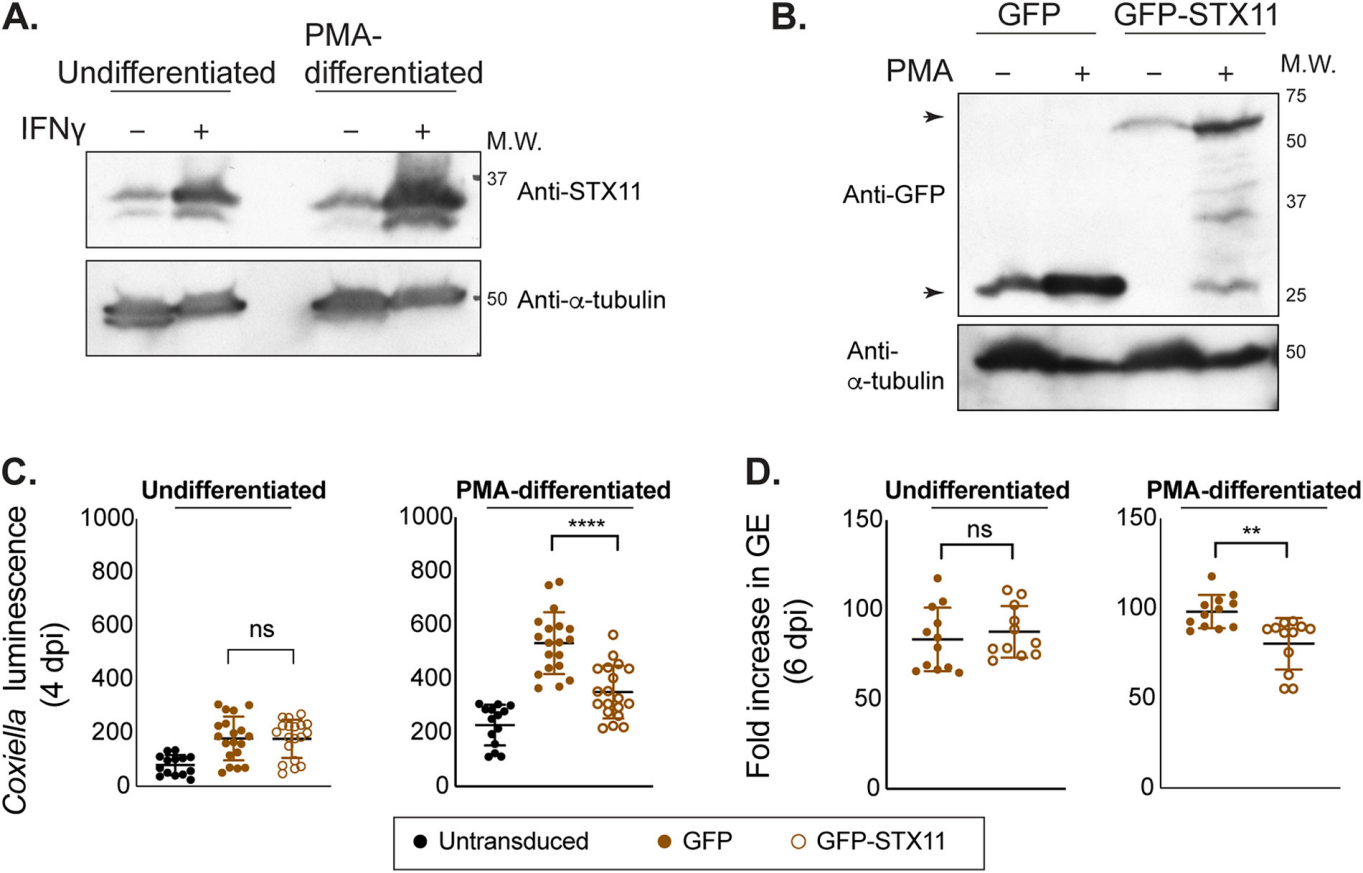
STX11 expression interferes with C. burnetii replication in macrophages. (A) Endogenous STX11 expression in undifferentiated or PMA-differentiated THP-1 cells treated with IFNγ were examined by western blotting. (B) THP-1 cells expressing GFP-STX11 or GFP alone were treated with PMA as indicated, and expression of GFP-tagged proteins was examined by western blotting. Arrowheads indicate GFP-only and GFP-STX11 fusion proteins at the expected molecular weights. (C and D) THP-1 cells, transduced and differentiated as indicated, were infected with C. burnetii *lux* at MOI of 25, and luminescence was measured at 4 dpi (C), or cells were infected with wt C. burnetii, and bacterial GE were measured at 6 dpi by qPCR and normalized to that at 1 dpi (D). All biological replicates from 3 independent experiments are represented in dot plots (C and D). Statistical significance was measured by *t* tests (**, *P* < 0.01; ****, *P* < 0.0001) comparing C. burnetii replication in GFP-STX11- and GFP-expressing cells (C and D).

Because macrophages are the primary cells that support C. burnetii replication during human infection, the role of STX11 in cell autonomous defense was investigated using the human monocytic cell line THP-1. Treatment of THP-1 cells with phorbol 12-myristate 13-acetate (PMA) induces differentiation of these immature monocytic cells into macrophage-like cells. Immunoblot analysis showed that STX11 is upregulated after IFNγ treatment in both undifferentiated and PMA-differentiated THP-1 cells (Fig. 5A). THP-1 cells that ectopically produce either GFP alone or GFP-STX11 were infected with C. burnetii to test whether elevated expression of STX11 interfered with intracellular replication (Fig. 5B). The PMA-treated macrophages producing GFP-STX11 were less permissive for C. burnetii replication than control cells producing GFP alone, as determined by measuring bacterial luminescence after 4 days of infection (Fig. 5C). Inhibition of C. burnetii replication in these cells was confirmed by GE measurements (Fig. 5D). No significant difference in C. burnetii replication was detected in undifferentiated THP-1 cells producing GFP-STX11, which could indicate a difference in the functionality of this antimicrobial pathway in monocytes compared to macrophages or could be related to higher levels of GFP-STX11 protein being detected in the PMA-treated cells. These data confirm that STX11 is important for a defense pathway that limits C. burnetii replication in human cells.

## DISCUSSION

This study identified STX11 as a host factor important for restriction of C. burnetii intracellular replication. Notably, knockdowns in several IFNγ-induced genes typically involved in host defense against intracellular pathogens, including *GBP1*, -*2*, and -*5* (encoding IFNγ-induced guanylate-binding proteins), *CYBB* (encoding 91-phox), and *NOS2* (encoding inducible nitric oxide synthase [iNOS]), did not enhance C. burnetii replication in HeLa cells, which may indicate that C. burnetii has mechanisms to evade these host defense mechanisms. In particular, GBPs are often recruited to pathogen-containing vacuoles and restrict pathogen replication by promoting cell-autonomous defense pathways that include vacuole rupture, activation of inflammasomes, and autophagy (46–49); however, efficient recruitment of GBP1 to the CCV has not been observed (our unpublished data).

STX11 is a well-characterized component of the machinery that promotes the fusion of lysosome-related organelles (LROs) with the plasma membrane to mediate release of lytic cargo into the immunological synapse, which is an important function in cytotoxic T lymphocytes and natural killer cells (50–53). Mutations in STX11 are associated with impaired exocytosis of lytic granules and predispose humans to an immune disorder called familial hemophagocytic lymphohistiocytosis subtype 4 (FHL-4) (51, 54). Further studies have provided evidence for uncontrolled macrophage activation, cytopenia, hepatosplenomegaly, and elevated levels of multiple proinflammatory cytokines in the serum in FHL-4 patients, which cannot be accounted for by an exocytosis defect (55) and suggests other roles for STX11. The early and aggressive onset of symptoms in primary hemophagocytic lymphohistiocytosis (HLH), as well as patients with macrophage activation syndrome (MAS, or acquired HLH), is predominantly triggered by infections (56), indicating a link between infection, immunity, and disease progression.

There are several attributes of STX11 that suggest a role in host defense and antimicrobial responses. STX11 is abundantly expressed in immune cells, is upregulated by immune activation through LPS and IFNγ, and is enriched in the phagosomes of IFNγ-treated cells (38, 45, 53, 57). In addition, STX11 expression is very high in placenta, lung, and heart, which is a tissue distribution that aligns closely with the tropism of C. burnetii and many other intracellular pathogens (38, 44, 58). Findings presented here support a nonlymphocyte role for STX11 in controlling intracellular bacterial infection.

STX11 interferes with C. burnetii replication at uninduced levels of expression and as an induced effector of IFNγ-mediated cell-intrinsic defense. The contribution of STX11 in restricting C. burnetii was validated by reconstituting STX11-deficient cells with STX11. The deficiency in STX11 did not measurably affect pathogen uptake or initial events postinternalization. Differential staining to measure extracellular and intracellular C. burnetii in wild-type and STX11-deficient cells did not reveal any uptake differences (data not shown). *Coxiella* luminescence that measures bacterial metabolic activity inside cells were not significantly different in wild-type and STX11-deficient cells at early times postinfection (Fig. 2B). The difference in C. burnetii replication in STX11-deficient cells was observed at roughly 3 to 5 days postinfection (dpi), which indicates that STX11 function interferes with C. burnetii replication at a later stage of infection (Fig. 2B).

Ectopic production of STX8 was observed to phenocopy ectopic production of STX11 with respect to limiting C. burnetii replication. STX8 is a SNARE involved in late endosome-lysosome fusion and exocytic secretion of granules in platelets, which are functions that are similar to those of STX11 (36, 37, 59–62). STX11 and STX8 have been found to be the most enriched SNAREs on phagosomes in IFNγ-activated macrophages implying a role in cell-autonomous immunity (57). SNARE-mediated fusion is driven by the formation of a 4-helix bundle between opposing membranes. Q-SNAREs (SNARE that contributes a conserved glutamine residue to the SNARE helical bundle) on donor membranes contribute 3 helixes (Qabc helixes) that typically interact with a helix on an R-SNARE (SNARE that contributes a conserved arginine residue to the SNARE helical bundle) on the acceptor membrane (24, 63, 64). STX11 is predicted to be a Qa SNARE that interacts with the Qbc SNARE SNAP23 and the R SNARE VAMP8 to mediate exocytosis of lytic granules (38, 52, 65). Importantly, GFP-STX11 produced in HeLa cells coimmunoprecipitated SNAP23 and VAMP8, suggesting that this SNARE bundle is generated in these cells (Fig. S3A and B). The proteins Vti1B (Qb SNARE) and STX8 (Qc SNARE) were also found in a complex with GFP-STX11, which suggests that an alternative pairing of SNAREs could mediate a cell-autonomous pathway that restricts C. burnetii replication. Previous data have implied that STX11 interacts with Vti1B to modulate the formation of functional SNARE complexes containing STX6 or STX8 to modulate exocytic and endocytic fusion pathways (61). Thus, STX11 can functionally interact with multiple SNAREs to modulate different cellular fusion events. Further, detection of STX11-interacting SNAREs in cells that have not been activated with IFNγ or infected with C. burnetii (Fig. S3A and B) suggests a homeostatic role for STX11. Enhanced STX11 expression after IFNγ stimulation suggests that the homeostatic role for STX11 is augmented during immune activation. Additional studies are necessary to determine how STX11, and potentially STX8, function in the pathway that suppresses C. burnetii replication.

The association of STX11 on the CCV suggests a direct role for this SNARE in mediating fusion of vesicles with the vacuole. GFP-STX11 puncta that colocalized with LAMP1 were observed on the CCV, which indicates delivery of GFP-STX11 to the limiting membrane of the CCV. These puncta could represent enrichment of STX11 at areas where vesicles displaying STX11 fuse with the CCV membrane. In comparison, a t-SNARE resident on the lysosome-derived CCV, such as STX7, displayed a more uniform distribution (66) (Fig. 4A and Fig. S4).

One possible role for STX11, which would be consistent with the localization of STX11 to the CCV, would be to promote the fusion of vesicles containing antimicrobial cargo with the CCV. This hypothesis would be consistent with STX11 mediating the fusion of LROs with the plasma membrane. LROs contain cargo that enhances microbial killing, such as lytic enzymes and antimicrobial peptides. One possibility is that STX11 is targeted to different subcellular organelles in different cell types to mediate fusion of LROs with either the plasma membrane or pathogen-containing vacuoles.

Intracellular bacterial pathogens employ multiple mechanisms to subvert SNARE functions to modulate organelle biogenesis and vesicle fusion in host cells. Effector proteins produced by bacterial pathogens such as *Legionella* and Chlamydia have been shown to function as SNARE mimics (67, 68) and enzymes that mediate SNARE or synaptotagmin cleavage (69, 70). Pathogens can manipulate recruitment of SNAREs to the pathogen-containing vacuoles to modulate fusion of the vacuole with host organelles (71). Mycobacterium tuberculosis actively delays the recruitment of syntaxin 8 to the vacuole in which it resides, whereas the bacterial effector protein SipA, produced by Salmonella enterica serovar Typhimurium, mimics STX8 (72, 73). Thus, it is conceivable that C. burnetii has evolved effector-mediated mechanisms to partially counteract STX11 function. Indeed, many C. burnetii type IVB effectors localize to the CCV, have coiled-coil domains similar to the eukaryotic SNARE domain, and are important for C. burnetii replication (74). Overall, identification of STX11 and STX8 as host proteins that limit C. burnetii intracellular replication defines a cell-autonomous pathway that provides host defense against microbial pathogens, which makes these proteins potential targets for bacterial effector-mediated inhibition.

## MATERIALS AND METHODS

### Cell lines.

HEK293T, THP-1, HeLa 229, and CCL2 cells were from ATCC. All cells were maintained in Dulbecco’s modified Eagle medium (DMEM; Gibco catalog no. 11965-118) supplemented with 10% heat-inactivated fetal bovine serum (FBS), referred to as complete medium, at 37°C with 5% CO_2_. HeLa cells were maintained with DMEM containing 5% FBS during the course of infection. THP-1 cells were cultured in complete RPMI (RPMI 1640 medium with ATCC modification [catalog no. A10491-01] supplemented with 10% FBS and 50 μM β-mercaptoethanol).

### Coxiella burnetii strains.

Wild-type Coxiella burnetii Nine Mile phase II (NMII) (RSA439) and a strain that constitutively expresses luminescence were used as described earlier (34) (Table 1). C. burnetii strains were grown for 6 days in acidified citrate cysteine medium 2 (ACCM-2) at 37°C, 2.5% O_2_, and 5% CO_2_ as described elsewhere (75, 76). Bacterial cultures were centrifuged at 4,000 rpm and 4°C for 15 min, and pellets were resuspended in DMEM containing 5% FBS and sonicated for 10 min. C. burnetii genome equivalents were measured by quantitative PCR as described previously (8).

**TABLE 1:** C. burnetii strains used in this study

Strain	Antibiotic (concn [μg/mL])
WT NMII RSA439	None
C. burnetii *lux* (generated and gifted by Shawna C. Reed, Roy Laboratory, Yale University) (78)	Kanamycin (375)
WT NMII RSA439 expressing mCherry under the control of the CBU_0311 promoter, gifted by Paul A. Beare (78)	Chloramphenicol (3)

### Reagents and antibodies.

Black 96-well plates with clear bottoms, recombinant human IFNγ, and l-tryptophan were from Corning Costar (3904), Biolegend (570206), and Sigma-Aldrich (T0254), respectively. Trp was resuspended in tissue culture-grade water and used at 0.3125 mM. As listed in Table 2, siGENOME SMARTpool siRNAs (unless mentioned otherwise) and Dharmafect (T-2001) were obtained from Dharmacon. For transfection of plasmids (Table 3), TransIT-LT1 (Mirus Bio, MIR 2300) or Lipofectamine 2000 (Invitrogen, 11668027) was used.

**TABLE 2:** SMARTpool siRNAs from Dharmacon^*a*^

Pool catalog no.	Gene symbol	Gene ID	Accession no.	GI no.	Description
M-011057-00	IFNGR1	3459	NM_000416	4557879	Interferon gamma receptor 1
M-012713-00	IFNGR2	3460	NM_005534	47419933	Interferon gamma receptor 2
M-003146-02	JAK2	3717	NM_004972	13325062	Janus kinase 2
M-003145-02	JAK1	3716	NM_002227	102469033	Janus kinase 1
M-011704-01	IRF1	3659	NM_002198	4504720	Interferon regulatory factor 1
M-020858-02	IRF9	10379	NM_006084	82734235	Interferon regulatory factor 9
M-003543-01	STAT1	6772	NM_007315	21536299	Signal transducer and activator of transcription 1
M-003544-02	STAT3	6774	NM_213662	47458819	Signal transducer and activator of transcription 3
M-006423-03	CEBPB	1051	NM_005194	28872795	CCAAT enhancer binding protein beta
M-009240-01	NOS2	4843	NM_000625	24041028	Nitric oxide synthase 2
M-011021-01	CYBB	1536	NM_000397	163854302	Cytochrome *b*_245_ beta chain
M-005153-02	GBP1	2633	NM_002053	4503938	Guanylate binding protein 1
M-011867-00	GBP2	2634	NM_004120	38327557	Guanylate binding protein 2
M-028450-01	IRGM	345611	XM_001127260	113416797	Immunity-related GTPase M
M-014116-01	IFITM3	10410	NM_021034	148612841	Interferon-induced transmembrane protein 3
M-019469-01	STX11	8676	NM_003764	33667037	Syntaxin 11
M-008317-00	RAB20	55647	NM_017817	8923400	RAB20, member of the RAS oncogene family
M-028161-01	RAB43	339122	NM_198490	50234888	RAB43, member of the RAS oncogene family
M-010337-01	IDO1	3620	NM_002164	156071492	Indoleamine 2,3-dioxygenase 1
M-019310-01	IDO2	169355	NM_194294	148539553	Indoleamine 2,3-dioxygenase 2
M-013432-01	APOL6	80830	NM_030641	87162462	Apolipoprotein L6
M-017402-02	APOL1	8542	NM_003661	21735613	Apolipoprotein L1
M-007380-02	SLC11A1	6556	NM_000578	109255240	Solute carrier family 11 member 1
M-009553-00	RAB36	9609	NM_004914	31795534	RAB36, member of the RAS oncogene family
M-011002-01	C4A	720	NM_007293	67190747	Complement C4A
M-005197-00	TNFRSF1A	7132	NM_001065	23312372	TNF receptor superfamily member 1A
M-011511-04	SOCS1	8651	NM_003745	4507232	Suppressor of cytokine signaling 1
M-003502-01	ICAM1	3383	NM_000201	4557877	Intercellular adhesion molecule 1
M-012982-02	CLIC2	1193	NM_001289	66346732	Chloride intracellular channel 2
M-003729-03	PLSCR1	5359	NM_021105	10863876	Phospholipid scramblase 1
M-008322-01	WARS	7453	NM_004184	7710155	Tryptophanyl-tRNA synthetase
M-004202-01	NMI	9111	NM_004688	4758813	N-Myc and STAT interactor
M-005844-01	CTSS	1520	NM_004079	23110961	Cathepsin S
M-007516-01	SLC2A3	6515	NM_006931	5902089	Solute carrier family 2 member 3
M-007402-02	SLC16A1	6566	NM_003051	115583684	Solute carrier family 16 member 1
M-007453-00	SLC22A2	6582	NM_003058	23510411	Solute carrier family 22 member 2
M-007143-01	DTX3L	151636	NM_138287	31377615	Deltex E3 ubiquitin ligase 3L
M-018178-00	GBP5	115362	NM_052942	31377630	Guanylate binding protein 5
M-007831-01	CCL2	6347	NM_002982	56119169	C-C motif chemokine ligand 2
M-006022-01	PSMB8	5696	NM_148919	73747874	Proteasome subunit beta 8
M-011699-01	IRF8	3394	NM_002163	55953136	Interferon regulatory factor 8
D-001810-10	ON-TARGETplus Nontargeting Control				
L-003543-00-005 (ON-TARGETplus SMARTpool siRNA)	STAT1	6772			Signal transducer and activator of transcription 1

a CCAAT, Cytosine-cytosine-adenosine-adenosine-thymidine box motif; RAB, Ras-associated binding protein; TNF, Tumor necrosis factor.

**TABLE 3:** Plasmids used in this study

Plasmid	Notes	Antibiotic	Source
pEGFP-C1		Kanamycin	Clontech
pDNR-Dual-STX11		Ampicillin	Harvard PlasmID Database (ID HsCD00005628)
pEGFP-C1 STX7	Cloned h*STX7* at the SalI site in pEGFP-C1 by ligation-independent cloning	Kanamycin	This study
pEGFP-C1 STX8	Cloned h*STX8* at the SalI site in pEGFP-C1 by ligation-independent cloning	Kanamycin	This study
pEGFP-C1 STX11	Cloned h*STX11* at the SalI site in pEGFP-C1 by ligation-independent cloning	Kanamycin	This study
pEGFP-C1 VAMP8	Cloned h*VAMP8* at the SalI site in pEGFP-C1 by ligation-independent cloning	Kanamycin	This study
PX459	Sp-Cas9-2A-Puro and single guide RNA	Ampicillin	Addgene, no. 62988
pVSV-G	Lentiviral envelope plasmid	Ampicillin	Gift from Jorge Galán, Yale University
psPAX2	Lentiviral packaging plasmid	Ampicillin	Gift from Didier Trono (Addgene no. 12260)
plentiCas9-Blast		Ampicillin	Addgene, no. 52962
plentiGuide-Puro		Ampicillin	Addgene, no. 52963
pLenti-EGFP		Ampicillin	Gift from Brett Lindenbach, Yale University
pLenti-EGFP-STX11	Cloned *STX11* at the BsrG1 site by ligation-independent cloning	Ampicillin	This study
pLenti-EGFP-STX8	Cloned *STX8* at the BsrG1 site by ligation-independent cloning	Ampicillin	This study
pLenti-EGFP-STX7	Cloned *STX7* at the BsrG1 site by ligation-independent cloning	Ampicillin	This study
pLenti-EGFP-STX2	Cloned *STX2* at the BsrG1 site by ligation-independent cloning	Ampicillin	This study

### siRNA screen and luminescence measurement.

siRNAs were resuspended in 1× siRNA buffer and stored as 10 mM or 2 mM stocks at −20°C. Forty-eight hours prior to infection, HeLa 229 cells (10^4^ cells/well) were reverse transfected with 25 or 50 nM siRNA using Dharmafect (0.2 μL/well) in 96-well black, clear-bottom plates. As controls, cells were transfected with transfection reagent alone (mock transfection). HeLa cells were infected with C. burnetii *lux* at a multiplicity of infection (MOI) of 100, and 5 h later, Trp (0.3125 mM) was added. One hour after Trp addition, cells were left untreated or treated with 10 ng/mL of IFNγ. Cells were washed and replenished with fresh medium without IFNγ at 1 dpi, and luminescence values were measured on specific days postinfection as indicated using a Tecan Infinite M1000 plate reader. Peak C. burnetii luminescence values measured from untreated cells during the course of a 6-day infection period (4 dpi or as indicated) were normalized to 100%.

### Immunoprecipitation, mass spectrometry, and Western blotting.

HeLa CCL2 cells stably expressing GFP alone or GFP-STX11 were plated at 1.25 × 10^6^ in two 10-cm dishes each and infected with wt C. burnetii or left uninfected. Three days postinfection, medium was removed, and cells were washed with phosphate-buffered saline (PBS), lifted using cell lifters, and collected in 15-mL conical tubes with a 400 × *g* spin for 5 min at room temperature (RT). Supernatant was removed, and pellets were washed in 1 mL PBS again, transferred to 1.5-mL microcentrifuge tubes, and centrifuged at 2,000 × *g* for 5 min. Supernatant was discarded, and cell pellets were lysed in 1 mL buffer (50 mM Tris-HCl [pH 7.5], 150 mM NaCl, 1% Nonidet P-40, 0.5% deoxycholate, cOmplete Mini protease inhibitor tablet) and incubated on ice for 15 to 20 min. The lysate was clarified by a low-speed spin at 1,000 × *g* for 5 min at 4°C, and the resulting supernatant was separated into 2 microcentrifuge tubes, one for immunoprecipitate (IP) (950 μL) and another for lysate (50 μL) (for Western blotting). Lysate samples for IP were incubated with 15 μL anti-GFP antibody (Sigma-Aldrich, 11814460001) for 45 min to 1 h at 4°C. Protein G Dynabeads (Invitrogen, 10003D) were washed twice with lysis buffer, and 50 μL protein G agarose slurry was added to each lysate with antibody sample for overnight incubation at 4°C. Beads were separated using a Dynamag magnet, and flowthrough was removed. The beads were washed twice with wash buffer (50 mM Tris-HCl [pH 7.5], 0.25 M NaCl, 0.1% NP-40, 0.05% deoxycholate).

For mass spectrometry, the beads were additionally washed three times with 25 mM ammonium bicarbonate, resuspended in a 5 to 10× bead volume of a 1/500 dilution of trypsin (Promega V5113) in 25 mM ammonium bicarbonate, and mixed well. The samples were incubated overnight at 37°C on a thermomixer with gentle rocking. The supernatants and a first deionized-water wash of the beads were combined and saved. These samples were further acidified with formic acid at a final 0.5% concentration and submitted to the Keck Proteomics facility at Yale. Data were analyzed using Scaffold software. A list with exclusive spectral counts for SNARE and vesicle traffic-related proteins was compiled using the following parameters: protein threshold, 99%; peptide threshold, 95%; and minimum number of peptides, 1.

For Western blotting, after two washes with wash buffer, 2× Laemmli dye with β-mercaptoethanol (β-ME) was added to the beads and 4× dye to the lysate (lysate control for immunoprecipitation). The samples were incubated at 99°C for 10 min and loaded on to 10% SDS-PAGE gels. For Fig. 2A, 3A, and 5A and B and Fig. S2A, cells were directly lysed using the 1× blue loading buffer (Cell Signaling Technology, 7722). Proteins were transferred onto polyvinylidene difluoride (PVDF) membranes. The primary antibodies anti-STX11 (Millipore Sigma, ABS1033, or Thermo Fisher, PA5-50800), anti-α-tubulin (Sigma-Aldrich, T9026), anti-GFP (Sigma-Aldrich, 11814460001, or GenScript, A01388), anti-STX8 (R&D Systems, AF5448-SP), anti-VAMP8 (R&D Systems, AF5354-SP), anti-Vti1B (Proteintech, 14495-1-AP) were prepared typically at 1:1,000 in 5% bovine serum albumin (BSA) or 5% milk in 1× Tris-buffered saline–Tween (TBST) per the manufacturer’s recommendations. The secondary antibodies goat anti-rabbit/mouse/sheep/goat IgG conjugated to horseradish peroxidase (HRP) (Thermo Fisher Scientific, 65-6120 and 62-6520; Novus Biologicals, HAF016; Abcam, ab6741) were used at 1:5,000. To confirm knockdown of *STX11* by siRNA, cells were reverse transfected with 50 nM siRNA in 96-well plates and treated with IFNγ for 48 h, and cell lysates were prepared using 1× blue loading buffer and analyzed for STX11 protein levels by immunoblotting.

### Generation of STX11-deficient cells using CRISPR.

*STX11* gRNA oligonucleotides were designed using the web-based guide RNA design platform developed by the Feng Zhang lab (https://zlab.bio/guide-design-resources) and are listed in Table 4. gRNA oligonucleotides were annealed and cloned in plentiGuide-Puro and PX459 plasmids at BsmBI and BbsI sites, respectively, according to protocols developed by the Zhang lab (http://www.addgene.org/crispr/zhang/) (77). For each cell line, at least two independent clones were used to determine the effect of STX11 deficiency on C. burnetii replication.

**TABLE 4:** Oligonucleotides used in this study

Primer or oligonucleotide	Sequence	Description or purpose
*STX11* F	CACCGTCTCGAACACGATGTCCTCG	gRNA oligonucleotide used for cloning in plentiGuide-puro and PX459
*STX11* R	AAACCGAGGACATCGTGTTCGAGAC	gRNA oligonucleotide used for cloning in plentiGuide-puro and PX459
pEGFP-C1-*STX11* F	GATCTCGAGCTCAAGCTTCGAATTCTGCAGTCGACATGAAAGACCGGCTAGCAG	Cloning h*STX11* in pEGFP-C1 vector at SalI by ligation-independent cloning
pEGFP-C1-*STX11* R	GATCCGGTGGATCCCGGGCCCGCGGTACCGTCGACCTACTTGAGGCAGGGACAG	Cloning h*STX11* in pEGFP-C1 vector at SalI by ligation-independent cloning
pEGFP-C1-*STX7* F	GATCTCGAGCTCAAGCTTCGAATTCTGCAGTCGACATGTCTTACACTCCAGGAGTTGG	Cloning h*STX7* in pEGFP-C1 vector at SalI by ligation-independent cloning
pEGFP-C1-*STX7* R	GATCCGGTGGATCCCGGGCCCGCGGTACCGTCGACTCAGTGGTTCAATCCCCATATGAT	Cloning h*STX7* in pEGFP-C1 vector at SalI by ligation-independent cloning
pEGFP-C1-*STX8* F	GATCTCGAGCTCAAGCTTCGAATTCTGCAGTCGACATGGCACCGGACCCCT	Cloning h*STX8* in pEGFP-C1 vector at SalI by ligation-independent cloning
pEGFP-C1-*STX8* R	GATCCGGTGGATCCCGGGCCCGCGGTACCGTCGACTCAGTTGGTCGGCCAGAC	Cloning h*STX8* in pEGFP-C1 vector at SalI by ligation-independent cloning
pEGFP-C1-*VAMP8* F	GATCTCGAGCTCAAGCTTCGAATTCTGCAGTCGACATGGAGGAAGCCAGTGAAGG	Cloning h*VAMP8* in pEGFP-C1 vector at SalI by ligation-independent cloning
pEGFP-C1-*VAMP8* R	GATCCGGTGGATCCCGGGCCCGCGGTACCGTCGACTTAAGAGAAGGCACCAGTGGC	Cloning h*VAMP8* in pEGFP-C1 vector at SalI by ligation-independent cloning
*dotA* F	GCGCAATACGCTCAATCACA	qPCR for C. burnetii GE
*dotA* R	CCATGGCCCCAATTCTCTT	qPCR for C. burnetii GE

**(i) HeLa 229 cells.** HeLa 229 cells stably expressing Cas9 were made by lentiviral transduction of HeLa 229 with plentiCas9-BLAST (Table 3) and selection with blasticidin (6 μg/mL), followed by clonal isolation. Cas9-expressing cells were left untransduced or transduced with pLentiGuide-puro, which expresses *STX11* gRNA (Table 3) or does not include a cloned gRNA (empty vector [EV]). At 48 h postransduction, cells were replenished with complete medium for a day, and then 2 μg/mL puromycin was added. Once the cells were stable in puromycin, they were clonally isolated to identify a clone that does not express STX11 protein by Western blotting.

**(ii) HeLa CCL2 cells.** HeLa CCL2 cells were transiently transfected with the PX459 plasmid, which expresses *STX11* gRNA (Table 3), using TransIT-LT1 per the manufacturer’s instructions. At 24 h posttransfection, cells were selected in puromycin (2 μg/mL) for 48 h prior to clonal isolation. Cells were not maintained in puromycin selection to avoid the stable integration of *Cas9* into the genome.

### Measurement of GE.

Cells (5 × 10^4^ per well for HeLa 229 or 2.5 × 10^4^ per well for HeLa CCL2) were plated in 24-well plates, 1 day prior to infection. Cells were infected with wt C. burnetii NMII at MOI of 100 and washed 1 dpi. At 1 and 7 dpi, supernatants were collected separately and combined with cells lysed with distilled water. Genomic DNA was extracted using the Illustra bacterial genomicPrep mini spin kit (catalog no. 28904259; GE) and genomic equivalents (GE) were quantified by qPCR using primers for the C. burnetii *dotA* gene (Table 4).

### Indirect immunofluorescence and live microscopy.

Cells (2.5 × 10^4^ or 5 × 10^4^) were plated on poly-l-lysine-coated coverslips placed in each well of 24-well plates, 24 h before infection. Cells were infected at MOI of 25 (HeLa CCL2) or 100 (HeLa 229) and washed at 1 dpi. Cells were fixed at 1, 4, or 5 dpi with 4% paraformaldehyde (PFA) for 20 min at RT. Coverslips were washed at least six times with 1× PBS, permeabilized, and blocked with 0.2% saponin, 0.5% BSA, and 1% (vol/vol) heat-inactivated FBS. Coverslips were stained with rabbit anti-C. burnetii (2), mouse anti-LAMP1 (clone H4A3; Development Studies Hybridoma bank at the University of Iowa), rabbit anti-LAMP1 (Cell Signaling Technology, 9091S), anti-GFP (Sigma-Aldrich, 11814460001), anti-STX8 (BD 611352), and DAPI (4,6-diamidino-2-phenylindole; Sigma-Aldrich, D9542) at 1:10,000, 1:250, 1:100, 1:100, 1:100, and 1:10,000, respectively. Goat anti-rabbit and -mouse IgG (H+L) highly cross-adsorbed secondary antibodies with Alexa Fluor 568, 488, or 647 conjugates were purchased from Thermo Fisher Scientific (Thermo Scientific, A32733, A32731, A11029, and A32727) and used at 1:2,000. Stained coverslips were mounted on glass slides using Prolong glass antifade reagent (Life Technologies).

For live microscopy, HeLa cells were plated at 0.2 million cells in 35-mm dishes (MatTek Corporation, P35G-1.5-14-C) or 0.05 million cells in 24-well plates with glass bottoms (NEST Scientific, 801006) and left overnight. Cells were transfected with pEGFP-C1 expressing GFP-tagged SNAREs using Mirus LT1 (MIR 2300), per the manufacturer’s instructions. At 6 h posttransfection, cells were infected with wt C. burnetii expressing mCherry controlled by the promoter of CBU_0311, at MOI of 100. Cells were washed six times with PBS after 18 h. Uninfected and infected cells were imaged at 2 or 3 dpi after staining with Hoechst (Thermo Fisher Scientific, H1399).

Images were captured with a Photometrics CoolSNAP EZ 20 MHz digital monochrome camera, Nikon Eclipse TE2000-S inverted microscope (Lumencor), and Nikon Plan Apo100x objective/1.4 numerical aperture as described earlier (34). Three-dimensional (3D) images were acquired using SlideBook software 6.0 (Intelligent Imaging Innovations), and z-stacks were deconvolved using nearest neighbors or constrained iterative methods. Deconvolved images were processed using Fiji, saved as TIFF files, resized, and labeled using Adobe Illustrator, and representative fields are shown. CCV area was measured by tracing the limiting membrane of the CCV using SlideBook software.

### Lentiviral expression of GFP and GFP-STX11 in HeLa cells.

The human *STX11* coding sequence (CDS) (gene ID 8676) was generously provided by Harvard PlasmID Database (ID HsCD00005628). The *STX11* CDS was cloned into an empty lentiviral vector, pLenti-EGFP, at the BsrGI site, C terminal to enhanced GFP (EGFP) but separated by a linker (GGGGSGGGGS), through ligase-independent cloning using the primers listed in Table 4. Briefly, 3 million HEK293T cells were plated per 10-cm dish and transfected with pLenti-EGFP (empty vector) or pLenti-EGFP-STX11 with pVSV-G and psPAX2, as listed in Table 3, using Lipofectamine 2000 (Invitrogen). Supernatants containing lentiviral particles were collected at 72 h posttransfection and filtered using a 0.45-μm low-protein-binding filter (Millipore, SLHA033SS). Lentivirus containing pLenti-EGFP or pLenti-EGFP-STX11 was used to transduce subconfluent HeLa cells by incubating cells with a 1:1 ratio of lentiviral medium and complete medium in the presence of Polybrene (Sigma H9268; 8 μg/mL). Transduced cells were selected using zeocin (200 μg/mL) for two rounds followed by, in some cases, fluorescence-activated cell sorting (FACS)-based enrichment of GFP-positive cells. Expression of GFP or GFP-STX11 was verified by Western blotting using anti-GFP antibody.

### Culture and lentiviral transduction of THP-1 cells.

To derive GFP-only- or GFP-STX11-expressing lentivirus for transducing THP-1 cells, supernatants from packaging HEK293T cells were concentrated by ultracentrifugation at 25,000 rpm at 4°C for 2 h using a 45 Ti rotor. Lentivirus-containing supernatants from ~5 or 6 dishes of HEK293T cells were concentrated, and visible pellets were observed. The postspin supernatant was discarded, and pellets were dissolved in complete RPMI to concentrate 7.5-fold. A total of 1.5 million cells of THP-1 cells in 500 μL medium were mixed with 2 mL concentrated lentiviral supernatant and 2 μg/mL Polybrene, placed in 6-well plates, and spun down at 2,000 × *g* for 45 min at 37°C. After the spin, cells were gently pipetted a few times to avoid cell clumping. After 2 days, the medium was replaced with fresh complete RPMI. After a day of recovery, medium was supplemented with 600 μg/mL zeocin, a concentration determined to be effective for THP-1 cells by kill curves. Antibiotic-stable cells were sorted for GFP^+^ cells using FACS.

### Infection of THP-1 cells.

Parental THP-1 cells or transduced, FACS-sorted GFP- or GFP-STX11-expressing THP-1 cells were plated at 6 × 10^4^/well (96-well plate) for the C. burnetii luminescence-based growth curve assay, 2 × 10^5^/well (24-well plate) for the C. burnetii GE-based growth curve assay, and 3.5 × 10^5^ to 4 × 10^5^/well (12-well plate) for assessment of protein expression. Cells were treated with PMA (124 ng/mL) for 24 h for differentiation into macrophages. PMA-containing medium was removed, and cells were replenished with fresh medium prior to infection. Cells were infected with wt C. burnetii or C. burnetii *lux* at MOI of 25. Overnight infection was followed by washing the cells and replenishing with fresh complete RPMI. Luminescence/GE-based growth curves were performed similarly to that described for HeLa cells. Where indicated, cells were treated with IFNγ (100 ng/mL) for 48 h.

### Data analysis and statistics.

Data were graphed and analyzed using Prism 9 software. Statistical significance was determined by *t* test or one-way or two-way analysis of variance (ANOVA) depending on the number of groups and comparison and Bonferroni *post hoc* tests using Prism 9.
