# Supplementary figures and images for: Syntaxin 11 Contributes to the Interferon-Inducible Restriction of Coxiella burnetii Intracellular Infection
Source: mBio. 2023 Feb 2;14(1):e03545-22. doi: 10.1128/mbio.03545-22 (PMC9972978; doi:10.1128/mbio.03545-22)

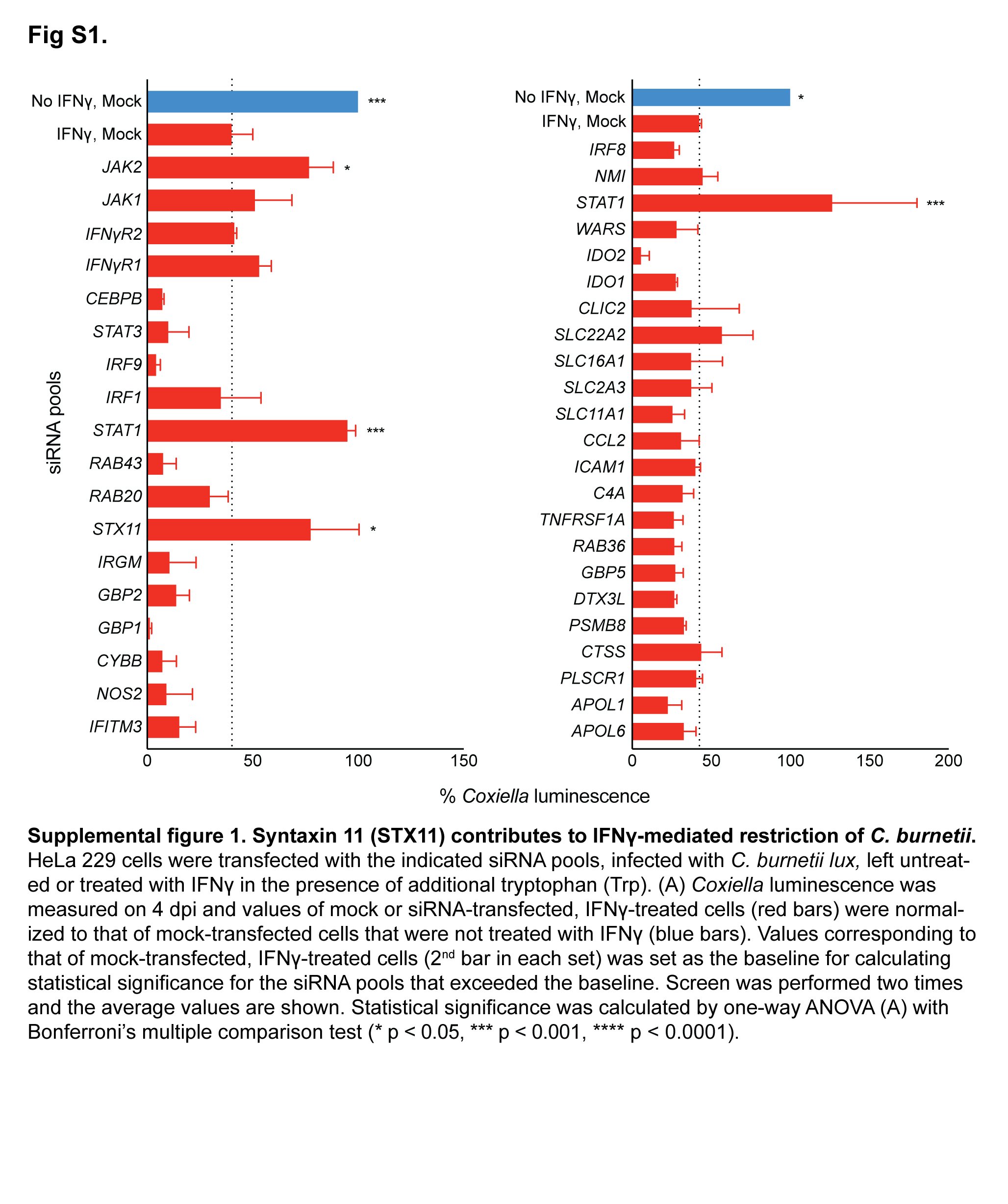

Supplement: FIG S1 [file mbio.03545-22-s0001.tif]

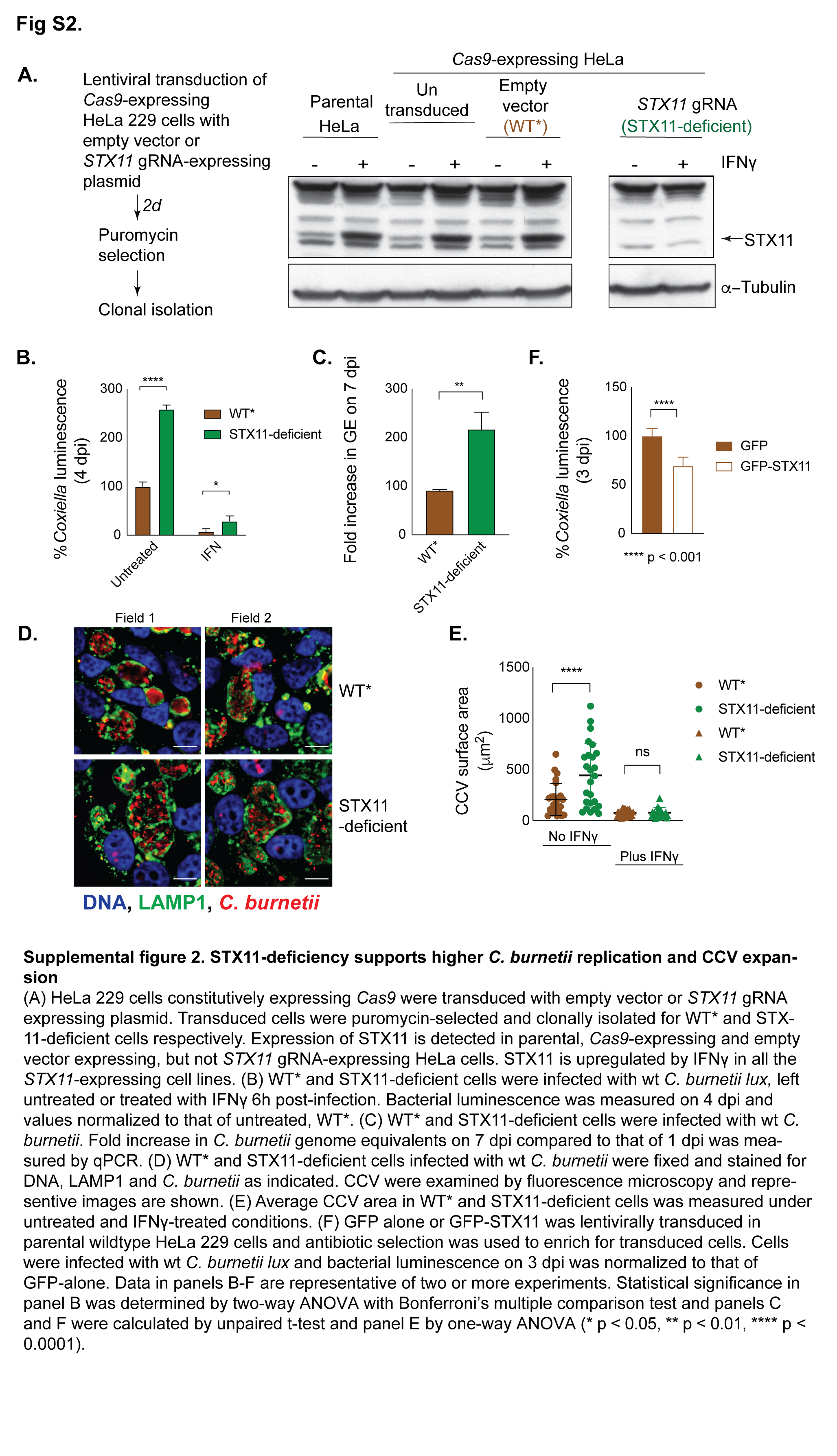

Supplement: FIG S2 [file mbio.03545-22-s0002.tif]

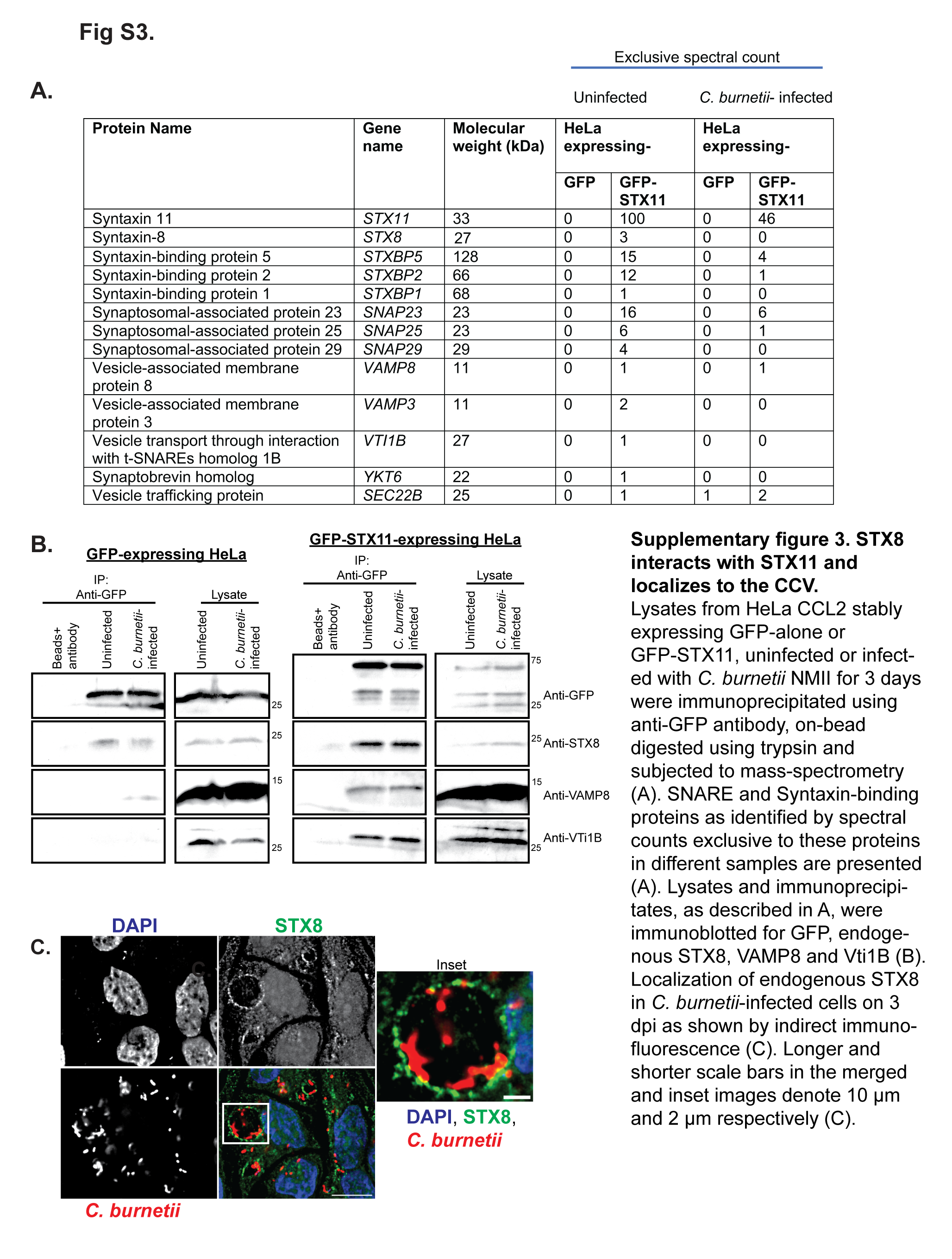

Supplement: FIG S3 [file mbio.03545-22-s0003.tif]

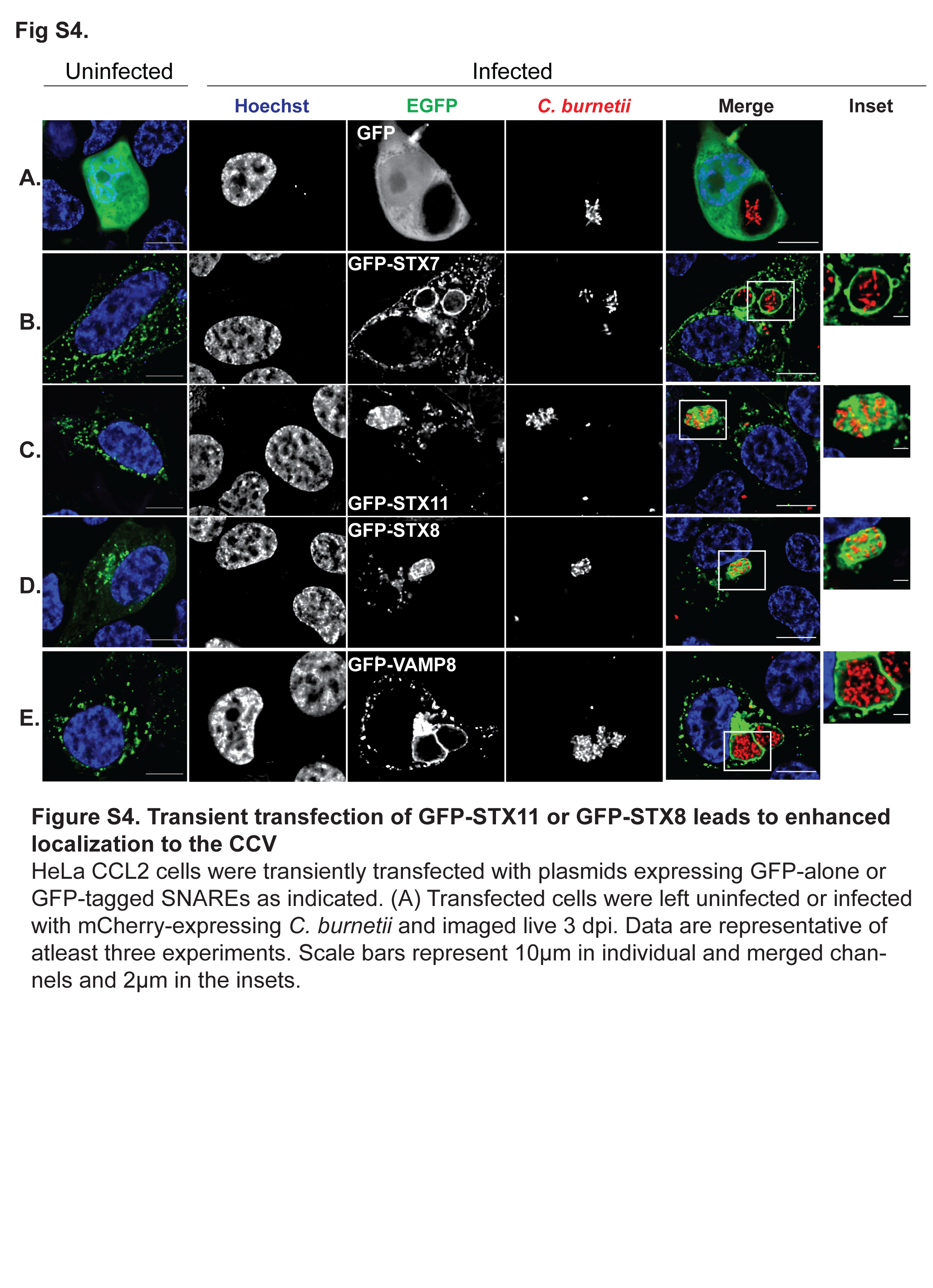

Supplement: FIG S4 [file mbio.03545-22-s0004.tif]
